# Cost-impact analysis of baroreflex activation therapy in chronic heart failure patients in the United States

**DOI:** 10.1186/s12872-021-01958-y

**Published:** 2021-03-26

**Authors:** John Bisognano, John E. Schneider, Shawn Davies, Robert L. Ohsfeldt, Elizabeth Galle, Ivana Stojanovic, Thomas F. Deering, JoAnn Lindenfeld, Michael R. Zile

**Affiliations:** 1 University of Rochester Medical Center, Rochester, NY USA; 2 Avalon Health Economics, Morristown, NJ USA; 3 Texas A&M University, College Station, TX USA; 4 CVRx Inc., Minneapolis, MN USA; 5 Piedmont Heart Institute, Atlanta, GA USA; 6 Vanderbilt University Medical Center, Nashville, TN USA; 7 Medical University of South Carolina, Charleston, SC USA

**Keywords:** Costs, Economic, Heart failure, Baroreflex activation therapy

## Abstract

**Background:**

The study evaluated the cost of baroreflex activation therapy plus guideline directed therapy (BAT + GDT) compared to GDT alone for HF patients with reduced ejection fraction and New York Heart Association Class III or II (with a recent history of III). Baroreflex activation therapy (BAT) is delivered by an implantable device that stimulates the baroreceptors through an electrode attached to the outside of the carotid artery, which rebalances the autonomic nervous system to regain cardiovascular (CV) homeostasis. The BeAT-HF trial evaluated the safety and effectiveness of BAT.

**Methods:**

A cost impact model was developed from a U.S. health care payer or integrated delivery network perspective over a 3-year period for BAT + GDT versus GDT alone. Expected costs were calculated by utilizing 6-month data from the BeAT-HF trial and existing literature. HF hospitalization rates were extrapolated based on improvement in NT-proBNP.

**Results:**

At baseline the expected cost of BAT + GDT were $29,526 per patient more than GDT alone due to BAT device and implantation costs. After 3 years, the predicted cost per patient was $9521 less expensive for BAT + GDT versus GDT alone due to lower rates of significant HF hospitalizations, CV non-HF hospitalizations, and resource intensive late-stage procedures (LVADs and heart transplants) among the BAT + GDT group.

**Conclusions:**

BAT + GDT treatment becomes less costly than GDT alone beginning between years 1 and 2 and becomes less costly cumulatively between years 2 and 3, potentially providing significant savings over time. As additional BeAT-HF trial data become available, the model can be updated to show longer term effects.

## Background

Chronic heart failure (HF) affects roughly 5.7 million in the U.S. and results in $30.7 billion in medical spending each year [1, 2]. In addition, each year in the U.S. there are more than 1 million HF-related hospitalizations [3]. Treatment of HF include pharmacological therapies, such as beta blockers, diuretics, ACE inhibitors, and angiotensin receptor-neprilysin inhibitors (ARNI) [4], and device-based therapies, such as implantable cardiac defibrillators and cardiac resynchronization therapy (CRT) [5]. For patients with more advanced stages of HF, treatment may include left ventricular assist devices (LVAD) and heart transplantation. A novel approach using baroreflex activation therapy (BAT) has shown promising results in clinical trials in these patients. BAT consists of device-based autonomic modulation, which generates a reduction of sympathetic outflow and an increase in parasympathetic activity, thereby rebalancing the autonomic nervous system [6]. This paper presents a cost impact model (CIM) of BAT + GDT versus GDT presented from the perspective of a U.S. health system or integrated delivery network. The model is based in part on the recently released 6-month clinical trial results associated with BAT administered via the BAROSTIM NEO System™ (CVRx, Inc., Minneapolis, MN) [7, 8]. The model also uses data abstracted from recent HF literature [9–12]. The working hypothesis is that BAT therapy is cost-saving; though it is a more resource-intensive in the short run, it results in better clinical outcomes over time, which in turn results in downstream cost savings for HF patients.

Several studies have demonstrated the safety and effectiveness of BAT in HF patients, as well as improved health-related quality of life (HRQoL) [7, 8, 13, 14]. BAT is delivered with an implantable device designed to modulate the body’s natural cardiovascular balance by sending communicative signals to the brain via an electrode attached to the outside of the carotid artery, which in turn activates the process of balancing sympathetic and parasympathetic activities to regain homeostasis [15]. The device is primarily intended for NYHA Class III or Class II (with recent history of Class III) patients with LVEF ≤ 35% and a NT-proBNP < 1600 pg/mL. In past clinical studies of HF patients, BAT has been attributed to improvements in HRQoL, activity tolerance, NYHA functional class and NT-proBNP levels [7, 8, 16, 17].

The safety and efficacy of BAT in subjects with systolic HF was further evaluated in the recent Baroreflex Activation Therapy for HF (BeAT-HF) trial [7, 8] Six-month data show that patients receiving BAT plus guideline directed therapy (BAT + GDT) significantly reduced NT-proBNP levels by 21% (*p* = 0.002) over a 6-month period from a median baseline value of 688 pg/mL, while patients with GDT alone showed an increase in NT-proBNP of 3% (*p* = n/s) from a median baseline value of 784 pg/mL, a 25% relative reduction (*p* = 0.004). Patients receiving BAT + GDT also saw significant improvements in functional capacity (*p* < 0.001) as measured by 6-min hall walk (6MHW). Consistent with the reduction in NT-proBNP and increase in 6MHW, patients receiving BAT + GDT experienced improvements in quality-of-life metrics including Minnesota Living with Heart Failure (MLWHF) and EQ-5D.

## Methods

**Fig. 1:**
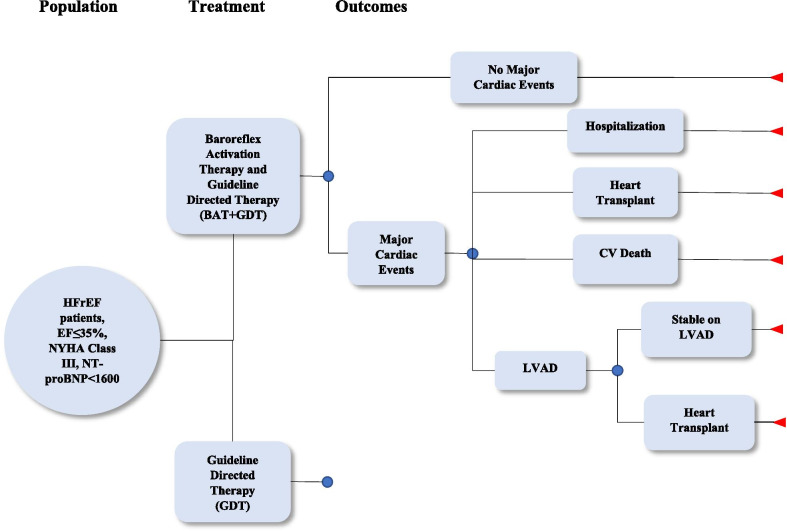
BeAT-HF decision tree model diagram

The CIM, developed in Microsoft Excel 2016, is structured to mirror the BeAT-HF trial comparing BAT + GDT to GDT alone over a simulated 3-year period, using 6-month data from the BeAT-HF trial combined with existing literature. Following a simple decision tree framework, as shown in Fig. 1, expected costs for each group are calculated by multiplying rates and probabilities (trial or literature-based) with cost estimates from the literature. Key cost-related outcomes considered were BAT-specific serious adverse events, HF and non-HF CV hospitalizations, HF medication utilization, and progression to LVAD or heart transplant. Calculations are performed at four time periods: 6 months (corresponding to trial data and including initiation of BAT) and years 1, 2, and 3 post-BAT.

Rates of BAT-specific serious adverse events, progression to LVAD and heart transplantation, and rates of CV non-HF hospitalization (GDT only) are based on 6-month BeAT-HF data. HF hospitalization rates for GDT are based on assumptions outlined within the statistical analysis plan for the BeAT-HF trial [18]. HF and CV non-HF hospitalization rates for BAT + GDT were calculated using a relative reduction estimated from changes in NT-proBNP levels (baseline to 6-month change) compared between in BAT + GDT and GDT alone. At 6 months, a 24.6% reduction in NT-proBNP between BAT + GDT and GDT alone was observed. A recent study demonstrated that a 25% reduction in NT-proBNP results in an approximately 25% relative reduction in CV mortality and HF hospitalizations (i.e., a hazard ratio of 0.75) [12].

### Clinical inputs

A summary of all clinical input parameters used in the model is shown in Table 1. HF medication utilization is based on trial data for the first 6-months of the model, and it was assumed that subjects taking medication remained on that medication throughout each time interval. While medication utilization may decline for an individual patient (e.g., due to poor compliance), overall medication utilization is likely to increase over time as disease progresses and new drugs become available. Medication use in the GDT group was assumed to increase 4% each time period [11]; given disease progression, medication utilization was assumed to increase for both groups but at a slower rate in the BAT + GDT group. Based on recent market growth trends, it was assumed that the rate of ARNI utilization would grow by 10% between each time period in GDT alone, and that the utilization rate of ARNI in BAT + GDT would also grow, but at a 20% slower rate than GDT alone. This is based on the BeAT-HF trial results suggesting lower utilization of treatments like ARNI among patients receiving BAT + GDT compared to patient receiving GDT alone.

**Table 1:** Probabilities and expected number of occurrences per patient in BAT + GDT vs. GDT alone groups: Treatment, events, and serious adverse events

Parameter	6 Month		1 Year		2 Year		3 Year	
	BAT + GDT	GDT Alone	BAT + GDT	GDT Alone	BAT + GDT	GDT Alone	BAT + GDT	GDT Alone
**Heart failure medications (a, b)**								
ACE/ARB (c)	0.97	1.07	1.95	2.13	3.90	4.44	5.85	6.84
Beta blocker	1.83	1.89	3.67	3.79	7.34	7.89	11.01	12.15
Digitalis	0.22	0.25	0.43	0.50	0.86	1.04	1.30	1.60
Diuretics	2.71	2.78	5.42	5.57	10.83	11.59	16.25	17.86
ARNI (d)	0.60	0.73	1.21	1.46	1.98	3.24	2.60	5.18
Ivabradine	0.07	0.07	0.13	0.14	0.26	0.30	0.39	0.46
MRA (e)	0.62	0.63	1.24	1.26	2.47	2.63	3.71	4.05
Other HF meds	1.05	1.31	2.09	2.61	4.18	5.44	6.27	8.38
Cardiovascular Hospitalization (non-HF) Rate, Relative Reduction = 25% (f)	0.08	0.10	0.15	0.20	0.3	0.40	0.45	0.60
Heart failure hospitalizations, relative reduction = 25% (f)	0.13	0.18	0.26	0.35	0.53	0.70	0.79	1.05
LVAD (g)	0.00%	1.64%	0.50%	3.25%	0.50%	6.40%	0.50%	9.44%
Heart transplant	0.00%	0.50%	0.50%	1.00%	0.50%	1.99%	0.50%	2.97%
**BAT-specific serious adverse events**								
Infections	2.61%							
Other (h)	0.87%							

*Notes and sources*: (a) HF medication usage based on BeAT-HF clinical trial data, values are expressed as the expected number of medications for patients within the given time frame; (b) Assumes medication use in BAT + GDT is proportional over time, while GDT alone usage increases an additional rate of 4% per year; (c) ACE = angiotensin converting enzyme inhibitors, ARB = angiotensin receptor blockers; (d) ARNI use in GDT alone is assumed to increase at a faster rate than that of other medication usage in GDT alone; (e) MRA = mineralocorticoid receptor antagonist; (f) estimates based on NTproBNP levels (see text); (g) Assumes that left ventricular assist device (LVAD) implantations do not increase in BAT cohort, but probability of LVAD in GDT cohort increases nearly linearly (see text); (h) “Other” includes the following BAT-specific serious adverse events, each of which occurred at a rate of 0.87%: Prolonged Intubation (requiring overnight stay); Antibiotic Allergic Reaction (BAT implanted at a later date); HF Exacerbation; Prolonged Stay due to Dizziness; Acute Respiratory Failure (consequence of anesthesia); Pneumonia Resulting in Intubation; Ischemic Stroke; Cranial Nerve Stimulation for Localized Neck Pain (341 days post implant)

Significant CV non-HF hospitalization (i.e., arrhythmias and other cardiac conditions) rates in GDT alone were based on the observed trial data and assumed to be constant over time, consistent with the assumptions in the BeAT-HF study (Table 1) [18]. The rates for HF and non-HF CV hospitalizations in BAT + GDT were estimated based on assumed relative reductions in hospitalizations attributable to BAT. HF hospitalization rates within BAT + GDT were based on anticipated reductions of HF hospitalizations due to the improvement in the 6-month NT-proBNP observed from BAT. Zile et al. examined the relationship between one-month changes in NT-proBNP and the hazard ratio (HR) of time to first CV mortality and HF hospitalization, and found that a 25% reduction in NT-proBNP was associated with a hazard ratio (related to reduction in CV mortality and HF hospitalizations) of 0.75 between subjects with at least a 25% reduction in NT-proBNP compared to those without a 25% reduction [12]. This hazard ratio was applied as a 25% relative reduction in the number of HF hospitalizations in BAT + GDT compared to the GDT alone rates. The reduction in CV non-HF hospitalizations between BAT + GDT and GDT was also assumed to be 25%, consistent with the methodology based on Zile et al.

Hospitalizations associated with LVAD and heart transplantation were estimated differently for BAT + GDT versus GDT alone. The probability of BAT + GDT receiving an LVAD or a heart transplantation were both assumed to be 0.50% in years post-baseline. Based on disease progression effects, it was assumed that patients receiving BAT + GDT would have a low and constant probability of LVAD or heart transplant within the first 3 years. Heart transplantation probabilities in GDT alone are calculated from the one-year probability of heart transplantation among HF patients reported in the literature (i.e., approximately 1.0% per year over 5 years) [10]. LVAD and heart transplantation were estimated for GDT alone by converting the initial probability into an instantaneous rate.

### Cost inputs

All cost inputs were adjusted to 2018 U.S. dollars (Table 2). Cost of BAT was provided by the manufacturer and includes the cost of the device and the associated implantation surgery. Annual GDT costs were based on the monthly whole acquisition medication costs for HF patients, which reflect approximate payer reimbursement amounts. Given the short time horizon, future costs are not discounted. The “other’ category includes a wide range of medications, such as amiodarone and hydralazine.

**Table 2:** Summary of cost inputs associated with clinical inputs, 2018 U.S. Dollars

Parameter	2018 $US
BAT	$34,500
**Heart failure medications, annual (a)**	
ACE/ARB (b)	$852.00
Beta Blocker	$34.20
Digitalis	$480.00
Diuretics	$144.00
ARNI	$6,109.44
Ivabradine	$5,309.04
MRA (c)	$503.76
Other HF meds	$1,299.32
CV non-HF hospitalization (d)	$6,731.68
Heart failure hospitalizations	$6,293.13
LVAD (e)	$186,954.96
Heart transplant	$173,846.71
**BAT specific serious adverse events (f)**	
Infections	$11,842.98
Prolonged intubation (requiring overnight stay)	$2,527.66
Abx allergic reaction (BAT implanted at later date)	$4,824.03
Heart failure exacerbation	$2,527.66
Acute respiratory failure (consequence of anesthesia)	$10,565.57
Pneumonia resulting in intubation	$5,775.02
Ischemic stroke	$16,635.63

Notes and sources: (a) based on estimated monthly costs for average recommended dosages, extrapolated to represent annual costs; (b) ACE = angiotensin converting enzyme inhibitors, ARB = angiotensin receptor blockers; (c) MRA = mineralocorticoid receptor antagonist; (d) average of all CV non-HF hospitalization (6 month data); CV non-HF hospitalization include the following: Cardiac Arrhythmias/Cardiac Arrest, Hypotension/Syncope, Myocardial Infarction/Angina; (e) LVAD = left ventricular assist device; (f) two other BAT specific serious events [Prolonged Stay due to Dizziness; and Cranial Nerve Stimulation for Localized Neck Pain (341 days post implant)] occurred, but were not associated with any additional costs

CV non-HF hospitalizations include cardiac arrhythmias, cardiac arrest, hypotension, syncope, myocardial infarction, and unstable angina. The differential costs of each of these events were not reported for two reasons: the cost differentials are very small and insignificantly different from one another; and as reported in Table 1, these events occur in very small numbers in the clinical trial population. The average CV non-HF hospitalization cost is based on two sources: for those events associated with a specific diagnosis-related group (MS-DRG), the mean 2016 national hospital cost was used (adjusted to 2018 dollars); alternatively, estimates were based on the literature. LVAD and heart transplant costs were based on Medicare rates associated with relevant MS-DRG and procedure codes, calculated as the sum of physician and anesthesiologist fees and expected LVAD readmission costs in addition to the cost of DRG 002 (heart transplant or implant of heart assist system without major complications).

## Results

Table 3 showcases the expected costs per patient for both BAT + GDT and GDT alone, broken down by year for the first 3 years. In general, over time the annual costs associated with BAT + GDT are falling, while those associated with GDT alone increase. This results in a widening gap in per year costs in favor of BAT + GDT.

**Table 3:** Yearly expected costs per patient by treatment and by time period, BAT + GDT vs. GDT alone, 2018 US Dollars (HF hospitalization rate relative reduction = 25%)

Time period (a)	BAT + GDT	GDT Alone	Difference
1 Year	$53,141	$28,093	$25,047
2 Years	$14,184	$21,049	$(6,865)
3 Years	$9,541	$31,602	($22,061)

(a) time period is marked by the implantation of the BAT device; 6-month data pertains to the BeAT-HF clinical trial, whereas years 1, 2 and 3 are based on extrapolations (see text)

In the short term, treatment with BAT + GDT is more costly than GDT alone due to implantation costs, but over time the differential reverses, and BAT + GDT becomes less expensive than GDT alone (Table 4). After six months, corresponding to clinical trial results, BAT + GDT is $29,526 more expensive per patient than GDT alone, a differential almost completely attributable to the costs associated with the BAT device and its implantation. The two treatment groups have equal predicted costs before year 3 but predicted costs at and after the 3-year mark are lower in BAT + GDT group compared to GDT alone.

**Table 4:** Expected costs per patient by treatment and by time period, BAT + GDT vs. GDT alone, 2018 US Dollars (HF hospitalization rate relative reduction = 25%)

Time period (a)	BAT + GDT	GDT alone	Difference
6 Months	$43,600	$14,074	$29,526
1 Year	$53,141	$28,093	$25,047
2 Years	$67,325	$58,485	$8,840
3 Years	$80,565	$90,086	($9,521)

(a) time period is marked by the implantation of the BAT device; 6-month data pertains to the BeAT-HF clinical trial, whereas years 1, 2 and 3 are based on extrapolations (see text)

**Fig. 2:**
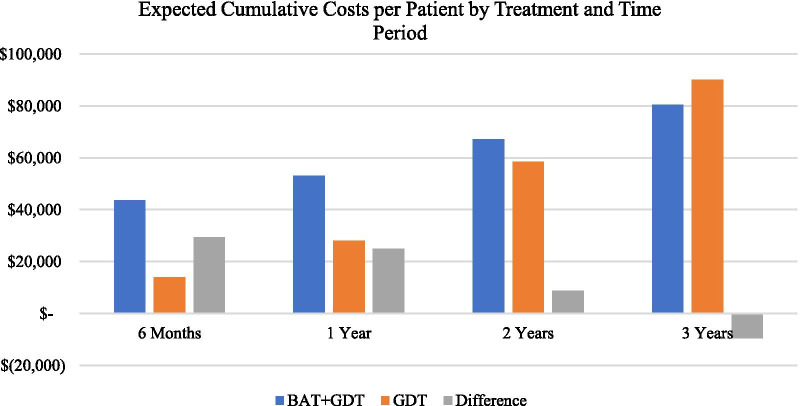
Expected cumulative costs per patient by treatment, 2018 US dollars

At 3 years, the predicted cumulative costs per patient for BAT + GDT was $80,565, while GDT alone costs were $90,086, representing a savings of $9521 using BAT + GDT. The initial cost of BAT made up roughly 43% of the predicted costs per patient for BAT + GDT at 3 years, making it one of the largest cost drivers within this group. Medication utilization was also a major contributor, consisting of 41% of predicted costs. Within GDT, medication utilization and the cost of LVAD were the largest contributors to costs at the 3-year mark, consisting of 63% and 20% of predicted costs, respectively. Costs savings for BAT + GDT accruing over time reflect the higher rates of disease progression, hospitalizations, and use of more resource-intensive treatments in GDT alone (Fig. 2).

The clinical data reported in the BeAT-HF trial showed significantly better clinical outcomes for the BAT + GDT patients across several measured endpoints. Using only NT-proBNP to predict hospitalizations, the model estimates substantially lower hospitalization rates in the years after BAT implantation (refer to Table 1), contributing to lower costs in the long run with BAT + GDT.

A one-way sensitivity analysis was conducted on each of the cost parameters. To assess the sensitivity of the findings to key variable, cost parameters were increased and decreased by 20% to measure impact on total BAT + GDT costs. Main cost drivers were the initial cost of BAT, the cost of ARNI and other medications, and the cost of LVAD. Any change in the cost of BAT showed a proportional change in the total costs of BAT + GDT, while GDT alone remained constant. At the 3-year mark, increasing the cost of BAT by 20% shows that savings with BAT + GDT fall to about $2621, while a decrease in the cost of BAT by 20% increases savings associated with BAT + GDT over GDT alone by $16,421. When LVAD costs are varied, BAT + GDT remains roughly unchanged; a 20% decrease in the cost of LVAD results in higher GDT alone costs compared to BAT + GDT by $6,178.

A 20% decrease in ARNI costs alone shows significant cost savings in GDT alone of greater than $6335, which is matched by a decrease in the costs in BAT + GDT of $3171. This result results in a difference of approximately $6358 in favor of BAT + GDT. Apart from the initial costs of BAT, GDT alone is more responsive to changes in the costs of model inputs. Given the lower complication and medication rates, costs within BAT + GDT vary less than those in GDT alone when subjected to increases and decreases in model input costs. The one-way sensitivity analysis showed that at the 3-year mark, there was some variability in which treatment becomes more cost effective, however, BAT + GDT remains less sensitive to most changes in model inputs. Given the lower rates of utilizations, complications, and hospitalizations, the difference in costs between the treatment arms will continue to grow in favor of BAT + GDT as time goes on and changes in model inputs will have less impact on results after 3 years.

## Discussion

Model results indicate that although there are larger initial costs associated with BAT + GDT, the economic benefits become evident in the longer run. Savings associated with BAT + GDT begin before 3 years post-implantation and continued to grow over time; HF patients with reduced ejection fraction and NYHA class III have a 3-year survival of 63.7%, implying that there will be considerable savings for most patients [19]. Model savings are attributable primarily to three factors. First, trial results associated with BAT + GDT imply that there will be fewer CV hospitalizations (e.g., fewer adverse events requiring hospitalization for HF) among BAT + GDT. Second, clinical benefits of BAT + GDT reported in BeAT-HF imply that, based on existing clinical practice guidelines, the need for LVAD and heart transplant may in some cases be delayed. Third, BAT + GDT requires an initial device and implantation cost, which means that the initial cost when spread over a 3-year period is negligible in terms of savings. It is also important to note that as more BAT clinical data become available, the methodology and results will be updated to give a more long-term (i.e., based on more than 6 months of trial data) representation of outcomes.

The findings are generally consistent with the working hypothesis. Without taking into consideration the improvements in quality of life, our analysis shows a great benefit to payers solely in terms of costs for BAT + GDT when compared with GDT alone. While there are currently no published cost-impact analyses for the treatment of chronic heart failure with BAT, a preliminary cost-effectiveness analysis in the German setting in patients with advanced chronic heart failure demonstrates that BAT is a cost effective treatment option, adding more evidence to support the use of BAT for the treatment of heart failure symptoms [20]. Although the model does not include a “budget impact” component which would account for “uptake” of BAT + GDT in a hypothetical payer population, given medical device adoption complexity and the time it takes to widespread utilization, we do not believe that adding BAT treatment option to an insurance plan would have a large budget impact in the first few years of the therapy’s existence on the market.

Several ad hoc sensitivity analyses and data checks were conducted for this analysis and not overly sensitive to variation in individual parameters. First, HF hospitalization estimates for time periods beyond 6 months were based on the BeAT-HF trial results pertaining to 6MHW. Though the prediction model was quite different (based on McCabe et al. [21]), the results were virtually identical. Second, model results were benchmarked with the existing literature. Within GDT alone, the model predicts a mean expected annual cost of $28,093 per person in the first year. In the U.S., annual HF costs are estimated to be about $26,000 (2018 dollars) but can also range as high as $40,000 depending on assumptions [22]. These ad hoc analyses suggest that the results of the CIM are consistent with prior literature and across methodologies.

There are some potentially important limitations to note. First, the model relies extensively on preliminary 6-month clinical data from BeAT-HF. These data, while showing significant results in key clinical endpoints, are still considered preliminary; the final release of the data will include longer intervals for most patients, which has the potential to alter some results reported within this study. Also, bias from using trial data may be present, as trial participants do not always represent patients treated, and benefits observed, in real world clinical practice. However, this is not expected to significantly limit the interpretation of the analysis, as the strength of preliminary trial data shows a robust relative benefit between the two randomized arms.

Second, the model assumes a 25% relative reduction in HF hospitalizations based on NT-proBNP levels to predict hospitalizations. Heart failure is a complex condition with many variables that impact outcomes, thus relying on NT-proBNP as a primary outcome indicator may be an oversimplification of reality. However, there is a substantial evidentiary base for NT-proBNP serving as a HF clinical endpoint predictive of future HF hospitalizations and health outcomes [23, 24]. Relative decreases in NT-proBNP have been shown to correlate with reduced all-cause mortality [25], heart failure hospitalization [12] and even left ventricular function [26]. The close association between reduced NT-proBNP and improved HF is sufficiently strong that management of individual HF patients according to their NT-proBNP levels has been proposed [27]. This is supported by a meta-analysis of 11 trials involving 2000 HF patients [28]. Combined, the studies demonstrate that such an approach significantly reduces all-cause mortality and hospitalization for HF or cardiovascular disease. To provide additional insight, prediction models based on the 6MHW showed remarkably similar results.

Third, the model does not consider other important health outcomes, such as “quality-adjusted” life years (QALYs). We suspect, however, that this is a limitation that may disproportionately affect GDT rather than BAT + GDT, as quality measures such as MLWHF and HF markers such as NT-proBNP and 6MHW show clear benefits associated with BAT + GDT (relative to GDT alone) in the BeAT-HF clinical trial. Thus, it is likely that a full consideration of these endpoints would result in higher QALYs within BAT + GDT group. Finally, results have not been discounted to reflect the time value of clinical benefits and costs. However, given the relatively short timeline considered, discounting is unlikely to have a meaningful impact on the results.

## Conclusion

Despite its initial device and implantation costs, BAT + GDT becomes the low-cost alternative treatment less than 3 years from implantation, based on observed and extrapolated clinical trial outcomes. These results are broadly consistent with other studies of BAT. The long-run impact (greater than 3 years) of BAT + GDT results in cost savings while resulting in improvements in a variety of outcomes measures. As such, the CIM suggests that BAT is a promising treatment option for patients with HF and a reduced LVEF who remain categorized in NYHA Class III or II (with a recent history of Class III) and have been on optimal standard-of-care pharmacological therapies. As BeAT-HF outcome data past 6 months becomes available, the methodology and results of this model will be updated to give a more long-term representation of outcomes between BAT + GDT and GDT alone.

## Data Availability

The datasets generated and/or analyzed during the current study are not publicly available because the BeAT-HF trial is still ongoing.

## Abbreviations

ARNI: Angiotensin receptor-neprilysin inhibitors
BAT: Baroreflex activation therapy
BAT + GDT: Baroreflex activation therapy plus guideline directed therapy
BeAT-HF: Baroreflex activation therapy for HF
CIM: Cost impact model
CRT: Cardiac resynchronization therapy
CV: Cardiovascular
HF: Heart failure
HR: Hazard ratio
HRQoL: Health-related quality of life
ICD: Implantable cardiac defibrillators
LVAD: Left ventricular assist device
LVEF: Left ventricular ejection fraction
MLWHF: Minnesota living with heart failure
QALY: Quality-adjusted life year
